# Wearable Technology in Gastroenterology: Current Applications and Future Directions

**DOI:** 10.3390/jcm14072403

**Published:** 2025-04-01

**Authors:** Keerthi D. Reddy, Saurabh Chawla

**Affiliations:** 1 Department of Medicine, Emory University School of Medicine, Atlanta, GA 30322, USA; kredd22@emory.edu; 2 Division of Digestive Diseases, Department of Medicine, Emory University School of Medicine, Atlanta, GA 30322, USA

**Keywords:** wearable, technology, gastrointestinal, biosensor, smartphone, heart rate variability

## Abstract

Advances in wearable technology have revolutionized healthcare by enabling the continuous monitoring of patients and personalized healthcare delivery. In the field of gastroenterology, the integration of wearable devices and smartphone applications represents a promising frontier. As technology continues to expand, understanding the current landscape and future directions of wearable technology in gastroenterology is essential for improving patient outcomes and clinical practice. **Background/Objectives**: Most review articles, thus far, regarding wearable technology in healthcare have been directed towards cardiovascular health. The purpose of this review is to explore the evolving role of wearable technology in the management of gastrointestinal disorders, focusing on remote patient monitoring and the use of smartphone applications. **Methods**: We conducted a search for studies on wearable technology and included the following search terms: wearable technology, gastroenterology, wearable device, smartphone, application, heart rate variability, biosensor, watch, patch. We included randomized controlled trials, prospective studies, and feasibility studies published from 2018 onwards. We excluded studies in pediatrics or those unrelated to GI disorders. **Results**: We found that using wearable devices and digital health management may be an effective way to monitor symptoms, reduce hospitalizations, and improve healthcare delivery in several gastrointestinal diseases such as inflammatory bowel diseases, motility disorders, liver diseases, etc. **Conclusions**: This review proposes that remote patient monitoring through wearable devices and digital health management via smartphone applications could reduce hospitalizations and empower patients, though challenges related to data security, accuracy, and integration with the electronic medical record must be addressed.

## 1. Introduction

The main ways in which wearable technology collects health data are through physical activity measurements, heart rate and rhythm sensors, sleep monitoring, and biochemical sensors. Physical activity is measured in a few different ways. One is through triaxial accelerometers, which measure linear acceleration along three different planes. The other is through the gyroscope, which measures angular motions. Lastly, global positioning systems (GPS) and barometry can be used to measure distance, altitude, stair counts, and falls. Using this information along with machine learning algorithms, human activities can be classified into walking, running, standing, and lying. Heart rate (HR) and rhythm is measured through photoplethysmography (PPG) and electrocardiography (ECG). The gold standard for monitoring HR is through measuring beat-to-beat intervals on an ECG machine. PPG uses changes in microvascular blood volume and translates those changes into pulse waves to measure HR. Most wearable devices use PPG to measure HR. Total sleep time is measured via similar methods, however it is more difficult to measure detailed sleep stages. Biochemical sensors measure body fluid electrolytes or specific biochemicals using electrochemical transducers. The accuracy of these biochemical sensors is affected by changes in skin temperature, skin contamination with dust or other substances, dried sweat, or other debris. They can be invasive or non-invasive. An example of an invasive biochemical sensor is a continuous glucose monitoring device (CGM). Non-invasive biochemical sensors are more practical to embed into other existing wearable technology [1,2].

Some wearable technology, such as the Apple^®^ (Cupertino, USA), Fitbit^®^ (San Francisco, USA), and Garmin^®^ (Olathe, USA) wrist wearables, have been approved by the Food and Drug Administration (FDA) to be used to monitor HR, physical activity, and sleep, based on results from prospective studies and clinical trial [1]. However, there are not many studies or clinical trials regarding other wrist wearables on the market or other non-medical grade wearable devices such as patches, rings, or biochemical sensors [1]. The CGM is the only biochemical sensor on the market that is FDA-approved for use at the time of this review. Further, wearable devices have been studied extensively for use in cardiovascular care, and many devices have been approved by the FDA for their use in such care [1]. They can be used for risk assessment via measuring physical activity levels and step counting. They can be used to assess atrial fibrillation by using PPG. They can also be used for cardiac rehabilitation by assessing activity levels while at home and providing telerehabilitation.

However, wearable devices have been studied to a lesser degree for applications in gastrointestinal care. The use of wearable technology in GI disorders could potentially be used for the early detection of flares, early intervention of cirrhosis decompensations, novel methods of assessing motility, and increasing monitoring of chronic conditions. The purpose of this review is to explore the evolving role of wearable technology in the management of GI disorders.

We searched PubMed and Embase for studies regarding wearable technology. We used the MeSH terms: “fitness trackers”, “gastrointestinal diseases”, and “gastroenterology”. We also used the following search terms: wearable technology, gastroenterology, wearable device, smartphone, application, heart rate variability, biosensor, watch, patch. We selected primary research and novel devices to review. We excluded systematic reviews and pediatric studies. We found that studies from 2018 onward had the most updated information and technology.

## 2. Wearable Technology for Inflammatory Bowel Disease (IBD)

Heart rate variability (HRV) refers to the fluctuation in the time interval between successive heartbeats. Analyzing HRV is a well-established non-invasive method to evaluate autonomic nervous system (ANS) function. Studies have shown that a low HRV is associated with a higher overall mortality. Thus, it is important to evaluate the role of HRV as it relates to various GI disorders. Hirten et al. found a significant association between HRV and ulcerative colitis (UC) flares [2]. Using the VitalPatch^®^ (VitalConnect, San Jose, CA, USA) device monitor, patients’ HRV was measured over 72 h every four weeks. They were also evaluated for UC flares using symptom assessments and laboratory values such as C-reactive protein (CRP) every 12 weeks and fecal calprotectin every 6 weeks. They found that a low HRV was associated with stress, and changes in the HRV from baseline were observed prior to development of UC symptoms or flares. DiJoseph et al. conducted a feasibility study in patients with UC using the WHOOP^®^ (Boston, MA, USA) wrist wearable [3]. They found the device easy for patients to use, and that patients reached out to providers with changes in their WHOOP^®^ metrics. These patients were then seen in the clinic prompting further testing, with serologic testing concerning for active inflammation. In these studies, HRV changes preceded the onset of symptoms. HRV may be a useful tool to guide the need for interventions and potentially preventing hospitalization. It also allows patients to have more ownership of their health and notify providers when there are changes in their HRV.

Another emerging method of predicting flares of IBD is through sweat sensors. Disease flares in IBD can occur without any definite precipitating factors, and currently there are no testing methods to track biomarkers in real time and predict the onset of a flare. Previously, continuous monitoring was thought to be unfeasible, as biomarkers, such as CRP and fecal calprotectin, require blood and fecal samples. Recently, certain biomarkers such as cytokines have been identified in sweat and could become important biomarkers to predict IBD flares [4]. In 2020, Jagannath et al. developed the first proof-of-feasibility report of continuously detecting cytokine and inflammatory markers in eccrine sweat using a wearable device [4]. Their device, called the SWEATSENSER, utilizes a replaceable sweat-sensing strip designed to detect specific biomarkers. The device is a wrist wearable, and it is integrated with an electronic reader that converts sensor impedance into concentrations of measured biomarkers in the sweat. Specifically, interleukin-1beta (IL-1b) and CRP are captured via monoclonal antibodies. They tested the SWEATSENSER in healthy subjects and found that they could detect IL-1b and CRP in the sweat continuously for 30 h over a range of 3 log orders. Jagannath et al. then showed that the SWEATSENSER device measured IL-6, IL-8, IL-10, and tumor necrosis factor-alpha (TNF-a) levels in the sweat accurately when compared to serum levels [5]. They also found that the levels of IL-8 were significantly different between a sick cohort and a healthy cohort. In 2024, Hirten et al. developed a sweat sensor adapted from SWEATSENSER called IBD AWARE [6]. They demonstrated that the IBD AWARE device can monitor sweat cytokines in subjects with IBD over 5 days when compared with daily checks of serum CRP and IL-6. They showed that TNF-a in sweat is elevated in patients with IBD flares compared to healthy controls. These studies show that sensors for cytokines in sweat could be an important, reliable way to monitor disease activity in IBD, but also in other inflammatory disease processes.

## 3. Wearable Technology for Motility Disorders

Diagnosing functional and motility gastrointestinal (GI) disorders can be challenging, particularly in the absence of invasive procedures [7]. In many cases, these diagnoses rely on subjective symptom assessments, unless the patient is evaluated at a specialized center. This approach, however, carries the risk of misdiagnosis and the potential for inappropriate treatment. Studies have suggested that electrical slow-waves in the gut muscle likely contribute to these disorders, but there are limited noninvasive diagnostic methods available, and many patients lack access to the specialized testing required for accurate diagnosis [7].

The electrogastrogram (EGG) is a noninvasive method that has been used previously to track electrical activity of the stomach. Yet, its use is uncommon due to technical obstacles like inconsistent outcomes from single-channel readings, signal disturbances from cardiac electrical activity, and anatomical differences, which hinder accurate interpretation and restrict extended monitoring periods [7]. Currently, EGG is not recommended for clinical use due to these limitations. In 2018, Gharibans et al. [7] developed a novel system of non-invasive mapping of gastric electrical activity. They created a peel-and-stick sensor array over the abdomen that allows for the recording and analysis of gastric electric activity across 24 h [7]. They were also able to identify and remove an artifact rather than removing the time in which artifact was seen. They validated this novel system against gastric manometry. In 2023, Gharibans et al. showed that this sensory array was tested in a cohort of healthy patients and successfully mapped gastric electric activity [8]. Vujic et al. developed a different novel system to measure gastric electric activity by creating a scuba-knit belt with recording electrodes placed in a standardized grid [9]. Such systems are limited by differences in anatomy across the population. Gharibans et al. [8] (2023) overcame this limitation by developing a companion smartphone application (app) to register the patient, assist with setup, and record data. The app guided optimal positioning of the array based on measurements between the xiphoid and umbilicus, the xiphoid and anterior superior iliac spine, and abdominal circumference [8]. These studies present an important development in the ability to conduct noninvasive, ambulatory gastric monitoring.

## 4. Wearable Technology for Cirrhosis

Patients with decompensated cirrhosis experience a significant number of hospitalizations, re-admissions, and cost to the healthcare system [10]. Bloom et al. [11] (2022) studied outcomes for patients with cirrhosis receiving the standard of care versus a novel telemonitoring system. They found that the most common reason for index hospitalization for patients with cirrhosis was ascites [12]. The most common reason for 30- and 90-day re-admissions was complications of cirrhosis, such as hepatic encephalopathy, ascites, gastrointestinal hemorrhage, or hepatorenal syndrome [10,12]. The ability to reduce re-admissions depends on whether the cause is preventable. Complications such as gastrointestinal hemorrhage or hepatic encephalopathy may not be preventable in the short term [12]. However, symptomatic ascites could theoretically be prevented with improved access to paracentesis.

Thus, Bloom et al. (2022) developed a telemonitoring system for patients with cirrhosis in which their weight is tracked remotely using Bluetooth-enabled scales and provides automated alerts to providers about weight changes [11]. The patients’ weight was used as a proxy for ascites volume. They studied this telemonitoring system against the standard of care, which was defined as routine outpatient follow-up or urgent care based on symptoms. Outcomes included hospitalizations, office visits, and paracenteses. They found that the telemonitoring system resulted in less re-admissions, more office visits, and more paracenteses. They estimated this was $167,500 less expensive than the standard of care for every 100 patients.

In 2019, Jansen et al. found that HRV monitoring using wearable devices was able to identify patients with cirrhosis at high risk of developing acute on chronic liver failure and death [13]. At two different clinic sites, 111 patients at risk of decompensated cirrhosis were given the Isansys Lifetouch wearable cardiac monitor and/or a Holter monitor. Both devices have the capability of recording HRV. HRV can be affected by numerous factors such as age, cardiac abnormalities, and respiration, and many patients with cirrhosis have autonomic dysfunction which can also affect HRV. Jansen et al. [13] (2019) attempted to overcome these factors using continuous, ambulatory measurement of HRV rather than its traditional measurement on EKG. They found that a reduced HRV correlated with severity of cirrhosis decompensation (*p* < 0.001). Reduction in HRV also inversely correlated with MELD and Child–Pugh scores (*p* < 0.0001 for both). Using HRV to monitor patients with cirrhosis could guide the need for early intervention (Table 1).

## 5. Remote Patient Monitoring (RPM) Using Smartphone Apps

Chronic GI disorders such as cirrhosis and IBD require close patient monitoring. Digital health platforms such as smartphone apps could greatly improve patient care. This method of patient monitoring has the potential to decrease emergency room visits, hospitalizations, and even face-to-face appointments.

Several studies have explored the use of apps for managing chronic GI diseases, demonstrating their feasibility and effectiveness in RPM and patient engagement. George and Cross [14] found that the myIBDcoach app reduced outpatient visits and hospitalizations for IBD patients compared to standard of care, though no significant differences were observed in disease activity. Therefore, the findings may not be applicable to patients experiencing active symptoms or severe disease. Coenen et al. [15] showed that the mynexuzhealth app effectively monitored IBD patients on stable therapy, detecting disease flares through integrated questionnaires, although compliance was moderate (52%). Similarly, McCombie et al. [16] highlighted that the IBDsmart and IBDoc apps (respectively, a symptom monitoring app and an at-home testing kit to monitor symptoms and fecal calprotectin) reduced outpatient visits, with good acceptability and adherence. Zhen et al. [17] found that the use of the healthPROMISE app led to fewer emergency visits and hospitalizations for IBD patients, though it did not significantly improve quality of care indicators.

Other studies focused on patient satisfaction and engagement. Dowd et al. [18] found high satisfaction with the MyHealthyGut app for managing celiac disease, particularly among newly diagnosed patients. Louissaint et al. [19] reported high acceptance of the EncephalApp from patients for cirrhosis management, but actual utilization was low (32%). Echarri et al. [20] demonstrated that a patient-administered Harvey–Bradshaw Index (HBI) app showed strong concordance with physician-administered HBI for Crohn’s disease, supporting its potential for remote monitoring. Chugh et al. [21] developed an integrated virtual care chat for IBD patients, achieving sustained engagement and high satisfaction, especially among patients with extraintestinal manifestations. Zand et al. [22] found that the UCLA eIBD app shows that the app is feasible for use by patients and providers in comprehensive IBD care by monitoring disease activity and delivering educational content.

In cirrhosis management, Kazankov et al. [23] (2023) found that the CirrhoCare^®^ (Stanmore, UK) app, which integrates daily monitoring of vital signs and two-way communication with physicians, was effective for managing decompensated cirrhosis, leading to fewer readmissions. This was a feasibility study in which the authors used feedback questionnaires in the CirrhoCare^®^ app (https://cirrhocare.com/) to assess the patients’ heart rate, blood pressure, weight, % body water, cognitive function using the CyberLiver^®^ Animal Recognition Test (https://www.cyberliver.com, 1 June 2024) app, self-reported well-being, and intake of food, fluid, and alcohol. There was two-way communication between the physician and patient through the app, including phone calls and text messages. The primary endpoint that was study was feasibility of remote monitoring (Table 2).

## 6. Challenges

Although wearable devices hold significant promise for revolutionizing healthcare, there are substantial barriers to their full integration into clinical practice. These challenges include concerns around data security and patient privacy, the accuracy of collected data, patient access to devices, and the responsibility of physicians to review and act on the data. To realize the potential of wearable devices in clinical settings, these issues must be systematically addressed.

Patient privacy is a central concern in the era of big data collection. Currently, data collected by wearable devices are often stored exclusively by the manufacturer, which raises questions about how these data can be securely shared for clinical use or research purposes, especially if patients consent. Devices that transmit patient health information (PHI) to electronic medical records (EMRs) must comply with existing regulations such as HIPAA (Health Insurance Portability and Accountability Act) and HITECH (Health Information Technology for Economic and Clinical Health Act). However, unless data are shared with an EMR or another covered entity, these regulations do not apply, creating a potential gap in data protection. A secure, standardized system is needed, that not only protects patient privacy, but also allows for data sharing across clinical and research settings. This may require amendments to HIPAA and HITECH or the creation of new regulations to keep up the pace with emerging technologies. Moreover, safeguarding wearable data from breaches is critical, as even de-identified data can potentially be re-identified through associated metadata [24]. Advanced cybersecurity measures must therefore be implemented to protect PHI.

The clinical utility of wearable devices in monitoring gastroenterological conditions depends on the accuracy of data collection and the effectiveness of the software used to process the raw data. Wearable devices are susceptible to motion artifacts, variability in skin contact, and other environmental factors, which can interfere with data accuracy. The performance of these devices is contingent on the quality of the algorithms used to filter out background noise, but the variability of physiological signals across different populations presents a challenge in developing standardized algorithms. Previous studies assessing the accuracy of wearable technology have yielded inconsistent results due to the lack of standardized experimental protocols [24]. To better manage the risks associated with wearable technology and biosensors, frameworks for evaluation, such as those proposed by Coravos et al. [25], are essential. Medical societies should also establish regulations and standards for the use of these devices. Additionally, it remains uncertain whether the data collected outside the clinical setting can be fully trusted. For example, in clinical environments, factors such as medication use and blood glucose levels are controlled during gastric emptying studies. However, in non-clinical settings, where these factors cannot be controlled, the data from an abdominal wearable device may be influenced by uncontrolled variables, potentially undermining its accuracy and reliability.

The integration of wearable devices into the clinical workflow also faces significant hurdles in terms of regulatory and practical issues. For instance, the current Meaningful Use criteria under HITECH, which guide the implementation of health technologies in clinical practice, do not yet include wearable devices and RPM as eligible tools for meeting these goals. This exclusion is partly due to the lack of robust clinical evidence demonstrating that wearable technologies and RPM offer superior outcomes compared to traditional care models [24]. Without strong evidence, recommending these devices to patients, many of whom would bear the cost, remains problematic. Most studies on wearables to date have been either feasibility studies or small-scale randomized controlled trials (RCTs), and further research is needed to confirm their efficacy. Furthermore, the responsibility for managing the data generated by wearables must be addressed. If wearable devices are integrated into the EMR, will the data be automatically reviewed by physicians, or will triage nurses be tasked with assessing its urgency? The influx of data could overwhelm physicians, particularly if it is transmitted frequently or without clear triage protocols. The logistics of incorporating wearable data into routine clinical workflows and ensuring timely, actionable review by healthcare providers must be thoroughly planned before widespread implementation can occur.

## 7. Conclusions and Future Directions

The demands on the healthcare system are continuously increasing, highlighting the need for innovative approaches to adapt to an evolving healthcare landscape. In this review we suggest that remote patient monitoring via wearable devices, alongside digital health management through smartphone apps, may offer a promising strategy to reduce hospitalizations and empower patients in managing their health. However, concerns around data security, data accuracy, and data delivery to the EMR must be addressed prior to their implementation.

These studies collectively underscore the potential of digital health and wearable technology to improve chronic GI disease management and enhance patient care. Further validation in larger RCTs is needed to evaluate whether the use of these technologies is truly superior to traditional care and assess the efficacy of novel wearable devices. Additionally, many of the discussed studies did not investigate patients with severe disease. The challenges remain in ensuring consistent use and optimizing the integration of such technology into routine care. Future studies should also focus on behavioral interventions to enhance adoption and assess physicians’ willingness to use the technology. Addressing these barriers is essential to ensure that wearable technology can be used safely.

## Figures and Tables

**Table 1:** Comparisons of wearable technologies. This table provides a comparison of the various technologies discussed in this article.

Device	Clinical Application	Patient Outcomes	Study Conclusions
VitalPatch (Hirten et al. [2])	HRV monitoring and UC flares	Measured HRV of patients with UC every 4 weeks for 72 continuous hours for the study duration of 9 months. HRV was analyzed by low frequency (LF) bands, high frequency (HF) bands, and LF to HF power (LFHF).	HRV reduction inversely correlated with inflammatory flare detected by FC (*p* < 0.001 for all 3 frequencies), and CRP (*p* < 0.001 for HF and LF; *p* = 0.09 for LFHF). HRV reduction significantly correlated with symptomatic flare detected by Simple Clinical Colitis Activity Index using only LFHF (*p* = 0.03).
WHOOP (DiJoseph et al. [3])	HRV monitoring and UC flares	Feasibility study in which patient used WHOOP to record symptoms. HRV was correlated to IBD related symptoms and monthly collections of SCCAI.	Two of the enrolled patients communicated concern for disease flare based on HRV and RHR changes detected by WHOOP which correlated with flare by serologic testing.
SWEATSENSER (Jaganath et al. [4])	Detect IBD biomarkers in sweat	Used a novel device to assess levels of IL-6, IL-8, IL-10, and TNF-a in sweat when compared with standard serologic testing	SWEATSENSER demonstrated a correlation of Pearson’s r > 0.98 for the study biomarkers when compared with the standard method.
IBD AWARE (Hirten et al. [6])	Detect IBD biomarkers in sweat	Used a novel device to assess sweat levels of CRP and IL-6 continuously compared to daily checks of serum CRP and IL-6 in hospitalized patients	Correlation between CRP measured in serum (lab-based) and sweat (from the IBD AWARE device) for the full data set of CRP values: R2 = 0.5278, serum CRP values of ≤20 μg/mL: R2 = 0.7108, serum CRP values of ≤10 μg/mL: R2 = 0.883. Correlation between IL-6 measured in serum (lab-based) and sweat (from the IBD AWARE device) for the full data set of IL-6 values: R2 = 0.601, serum IL-6 values of ≤20 pg/mL: R2 = 0.5732, serum IL-6 values of ≤10 pg/mL: R2 = 0.7231.
Novel EGG sensor (Gharibans et al. [8])	Mapping gastric electric activity	Tested a novel gastric mapping device tested in a cohort of 24 healthy subjects to define reliability and characterize features of normal gastric activity (30 m fasting, standardized meal, and 4 h postprandial).	Mean amplitude significantly increased during the 0–2 h postprandial period (*p* < 0.001 vs. fasted period) and during the 2–4 h postprandial period (*p* = 0.0001)
Novel EGG sensor (Vujic et al. [9])	Mapping gastric electric activity	Developed a novel scuba knit belt with a hydrogel electrode montage. Patients wore the belt in a free living environment during the recording.	The hydrogel electrode array captured gastric activity as well as artifact electrocardiogram activity.
Bluetooth scale and remote weight tracking (Bloom et al. [11])	Reduce cirrhosis admissions	Telemonitoring system that tracks patients’ weight remotely through Bluetooth-enabled scales and provides automated, early alerts to providers about weight changes. The outcomes included global costs, number of hospital admissions, office visits, and paracenteses.	Standard of care led to nine more admissions in a 6-month period than a telemonitoring intervention, while telemonitoring led to 28 additional outpatient LVPs in the same period. Cost of standard of care for 100 patients with cirrhotic ascites over a 6-month period is $1,221,500 and cost of care with a telemonitoring intervention was $167,500 less expensive.
Isansys Lifetouch or Holter monitor (Jansen et al. [13])	HRV and cirrhosis decompensations	Continuous HRV assessment in patients with cirrhosis in the outpatient setting, with acute decompensations, and with acute on chronic liver failure.	All cirrhosis patients had lower HRV compared to healthy subjects. Patients with acute decompensations had lower HRV compared to cirrhosis outpatients (*p* < 0.001), without significant differences in mean arterial pressure. Patients with acute on chronic liver failure had even lower HRV (*p* = 0.02).

**Table 2:** Comparison of smartphone applications. This table compares the various remote patient monitoring apps discussed in the article.

App	Clinical Application	Patient Outcomes	Study Conclusions
myIBDcoach (George and Cross [14])	IBD monitoring	RCT which compared telemedicine to standard of care. Patients were followed for one year after randomization.	Mean number of outpatient visits and telephone encounters were decreased in the telemedicine group (*p* < 0.0001 and *p* = 0.0003, respectively). Less patients were hospitalized in the telemedicine group (*p* = 0.046).
Mynexuzhealth (Coenen et al. [15])	IBD monitoring	Patients completed questionnaires on an app created by the study creators, and the data were sent to directly to the electronic medical record (EMR).	Nine patients triggered alerts for disease activity. For 8 of those patients, symptoms resolved spontaneously. One patient underwent endoscopy which confirmed IBD flare and treatment was changed.
IBDsmart and IBDoc (McCombie et al. [16])	IBD monitoring and FC detection	Patients used two apps created by the study creators (IBDsmart and IBDoc) to compare outpatient management vs. standard care.	Outpatient appointment numbers were reduced in smartphone app care (*p* < 0.001)
healthPROMISE (Zhen et al. [17])	IBD monitoring	Using the healthPROMISE app created by the study’s creators, metrics such as patient satisfaction, quality of life, and symptoms were collected and sent to the EMR.	The number of ER visits and hospitalizations compared to the prior year (without use of the app) significantly decreased from 25% of patients (8/32) to 3% (1/32) (*p* = 0.03).
MyHealthyGut (Dowd et al. [18])	Celiac disease management	Assess the effectiveness the app (MyHealthyGut) to help patients with celiac disease manage their disease and improve gut health.	Only participants the group given delayed access to use the app for a one-month period reported significant improvements in adherence to a gluten free (*p* < 0.001).
EncephalApp (Louissaint et al. [19])	Cirrhosis management	Assess patient acceptance of apps for management of cirrhosis using EncephalApp.	Intention of the patients to use the application was associated with perceived usefulness (β: 0.4, 95% CI: 0.3–0.5) and the presence of a caregiver (β: 1.1, 95% CI: 0.2–2.0). 71% agreed to download the app but actual usage was 32%.
HBI app (Echarri et al. [20])	Crohns Disease monitoring	Evaluate if a patient administered HBI on an app agrees with physician administered HBI in clinic.	All assessments showed a high percentage of agreement. Positive predictive value (PPV) for remission was 98.2%, and negative predictive value was 76.7%.
IBD app (Chugh et al. [21])	IBD monitoring	Evaluate the use of an app to improve IBD care and patient satisfaction and engagement. App data were directly sent to the EMR.	Patient satisfaction was moderately high with a median score of 8. Continuous engagement was significantly increased if patients reported presence of extraintestinal symptoms (7%, 95% CI: 0.01–0.14; *p* = 0.04).
eIBD (Zand et al. [22])	IBD monitoring	A feasibility study to assess patient satisfaction for IBD care using an IBD care app by patients and providers.	68% of patients were satisfied with communication using the app. 54% of patients reported improved perception of disease control and quality of life.
CirrhoCare (Kazankov et al. [23])	Cirrhosis monitoring	Feasibility to assess the management of acute decompensations of cirrhosis remotely using the CirrhoCare app.	Fifteen patients showed good engagement (≥4 readings/week), 2 moderate (2–3/week), and 3 poor (<2/week). Five patients had 8 readmissions for a median of 5 days, and none required hospitalization for >14 days. Outcomes require further validation in an RCT.

## Abbreviations

The following abbreviations are used in this manuscript:

PPG	Photoplethysmography
HR	Heart rate
ECG	Electrocardiography
CGM	Continuous glucose monitoring
FDA	Food and Drug Administration
IBD	Inflammatory Bowel Disease
ANS	Autonomic Nervous System
HRV	Heart Rate Variability
UC	Ulcerative colitis
CRP	C-reactive protein
IL-1b	interleukin-1beta
TNF-a	tumor necrosis factor-alpha
GI	gastrointestinal
EGG	electrogastrogram
EMR	electronic medical record
HIPAA	Health Insurance Portability and Accountability Act
PHI	Patient Health Information
HITECH	Health Information Technology for Economic and Clinical Health Act
RCT	Randomized Controlled Trial
